# Berberine prevents progression from hepatic steatosis to steatohepatitis and fibrosis by reducing endoplasmic reticulum stress

**DOI:** 10.1038/srep20848

**Published:** 2016-02-09

**Authors:** Zhiguo Zhang, Bo Li, Xiangjian Meng, Shuangshuang Yao, Lina Jin, Jian Yang, Jiqiu Wang, Huizhi Zhang, Zhijian Zhang, Dongsheng Cai, Yifei Zhang, Guang Ning

**Affiliations:** 1 Shanghai Institute of Endocrine and Metabolic Diseases, Shanghai Clinical Center for Endocrine and Metabolic Diseases, Department of Endocrinology and Metabolism, China National Research Center for Metabolic Diseases, Ruijin Hospital, Shanghai Jiao Tong University School of Medicine, Shanghai 200025, China; 2 Department of Molecular Pharmacology, Albert Einstein College of Medicine, Bronx, New York 10461, USA; 3 Department of Endocrinology, Yijishan Hospital of Wannan Medical College, Wuhu, Anhui Province 241000, China; 4 Department of Endocrinology, Xinhua Hospital, Shanghai 200092, China

## Abstract

The histological spectrum of nonalcoholic fatty liver diseases (NAFLD) ranges from hepatic steatosis to steatohepatitis and fibrosis. Berberine (BBR) is known for its therapeutic effect on obesity, hyperglycaemia and dyslipidaemia; however, its effect on NAFLD has yet to be thoroughly explored. Db/db mice and methionine-choline-deficient diet-fed mice were administered BBR via gavage. We found that BBR-treated mice were more resistant to steatosis in the liver than vehicle-treated mice and that BBR significantly reduced hepatic inflammation, fibrosis and lipid peroxides. The beneficial effect of BBR was associated with suppressing endoplasmic reticulum (ER) stress. Additionally, BBR decreased the free fatty acid-induced lipid accumulation and tunicamycin-induced ER stress in primary hepatocytes and hepatocyte cell lines. We demonstrated that BBR exhibited chaperone activity, reduced protein aggregation *in vitro* and alleviated tunicamycin-induced triglyceride and collagen deposition *in vivo*. Finally, we showed that BBR could reverse ER stress-activated lipogenesis through the ATF6/SREBP-1c pathway *in vitro.* These results indicated that BBR may be a new therapeutic strategy against hepatic steatosis and non-alcoholic steatohepatitis.

NAFLD is recognized as the leading cause of chronic liver disease in adults and children and a risk factor for a variety of metabolic diseases, including obesity, type 2 diabetes, and dyslipidaemia^1^. The prevalence of the disease has been dramatically increasing in western countries among obese and non-obese people and recently in Asia^2^,^3^. NAFLD encompasses a spectrum of liver injuries ranging from simple hepatic steatosis to non-alcoholic steatohepatitis (NASH) with or without fibrosis. NASH is defined by the presence of steatosis coexisting with hepatic inflammation and hepatocellular injury. Although simple steatosis has generally a mild prognosis, its progression to NASH can lead to liver fibrosis that may continue its course to cirrhosis and complications that include hepatocellular carcinoma, resulting in increased morbidity and mortality^4^.

The ideal treatments for NAFLD should not only reverse the accumulation of triglycerides (TG) in hepatocytes but also effectively suppress hepatic inflammation, thereby preventing simple steatosis from developing into NASH and fibrosis^5^. However, to date, no pharmacological treatment is approved for NAFLD/NASH, and the mechanisms underlying NAFLD pathogenesis remain unclear; the suggestion of healthy lifestyle modifications is the only remedy^6^.

*Coptis chinensis* has been used in traditional Chinese medicine (TCM) with a long history. Berberine (BBR) is a naturally occurring plant alkaloid present in *Coptis chinensis* and several other Chinese herbal medicines. BBR, which is commonly used for treating diarrhoea in China, is noted to have a glucose-lowering effect^7^,^8^. Our study has shown that BBR can reduce body weight by activating a thermogenic program in both the brown adipose tissue (BAT) and white adipose tissue (WAT) in db/db mice^9^. Kong *et al* reported that BBR may improve LDL working through a unique mechanism distinct from statins^10^. In a multi-centred, double-blinded clinical trial, our group demonstrated the beneficial effects of BBR on type 2 diabetes and hyperlipidaemia^11^. Moreover, intraperitoneal (IP) injection and oral gavage of BBR have been shown to alleviate TG deposition in the livers of obese mice and HFD-fed rats^12^,^13^. Recently, BBR has been reported to ameliorate NAFLD and related metabolic disorders in patients in a randomized, parallel-controlled, open-label clinical trial^14^. However, whether BBR has a beneficial effect on NASH and its molecular mechanisms remains to be determined.

To solve this problem, we evaluated the effects of BBR by using db/db mice and methionine-choline-deficient (MCD) diet-fed mice, which have both been previously shown to mimic humans with the similar character of liver steatosis, steatohepatitis and liver fibrosis, especially in the latter model. We examined whether BBR could work against the pathogenesis of NAFLD both *in vivo* and *in vitro*. We also elucidated that suppressing ER stress by using BBR is the central mechanism in improving NAFLD/NASH.

## Results

### BBR ameliorated liver steatosis and steatohepatitis in db/db mice

**Figure 1:**
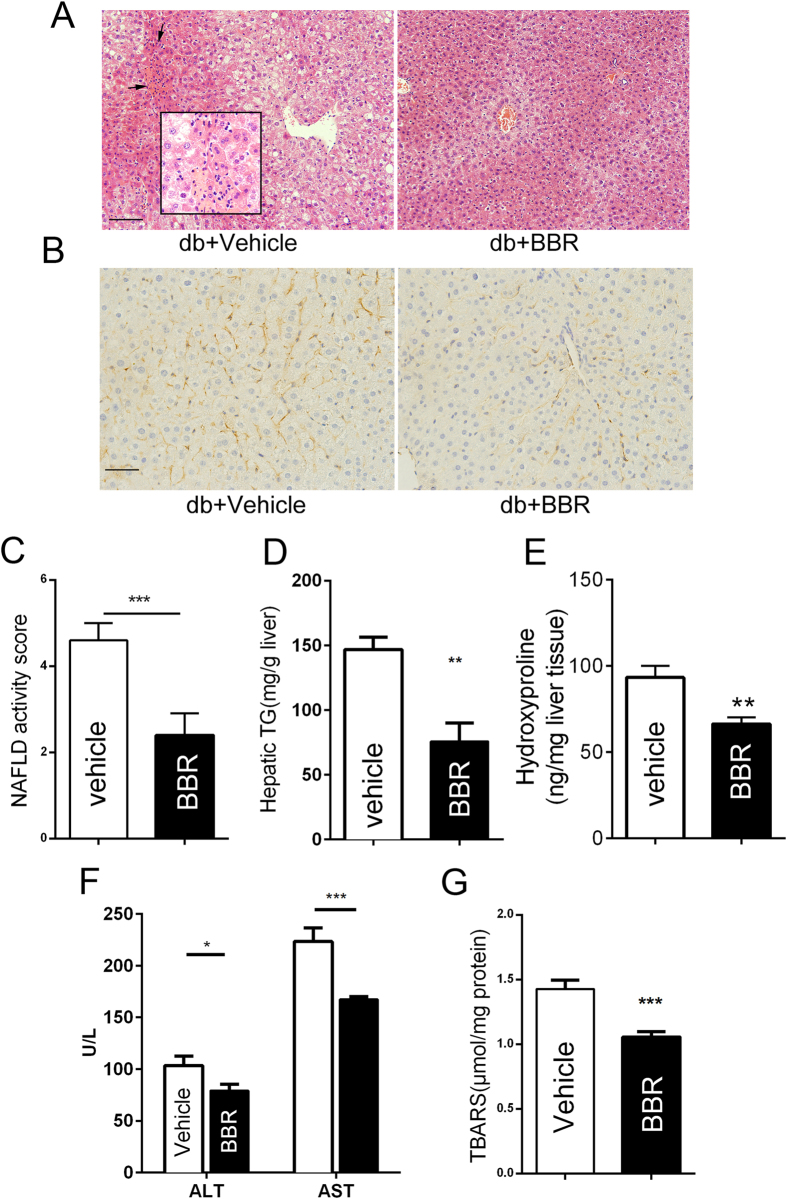
BBR-treated db/db mice are resistant to liver steatosis. Db/db mice were gavage treated using vehicle or BBR. (**A**) Histological analysis of haematoxylin-eosin-stained liver sections (200×), scale bar, 100 μm. The enclosed box in each panel shows the enlarged images (400×) and the black arrows indicate inflammatory foci. (**B**) Immunohistochemistry for α-SMA protein (brown stain) in liver sections (400×), scale bar, 50 μm. (**C**) Histopathology analysis was quantified by NAS. The values are the mean ± SEM, n = 4 or 3. (**D**) Quantification of hepatic TG content. (**E**) The hydroxyproline content was measured in the liver of mice. (**F**) Serum levels of ALT and AST. G: Lipoperoxides were measured as described in the Materials and Methods section. The data represent the mean ± SEM values. *P < 0.05, **P < 0.01, ***P<0.001, n = 7–8.

Our results demonstrated that BBR reduced body weight and ameliorated insulin resistance; thus, the disordered metabolic profiles were significantly improved (Supplementary Fig. 1A–F). Histological analysis using HE staining of the liver sections showed excessive lipid droplets and inflammatory foci within the lobule in db/db mice, which were alleviated by BBR after 5 weeks of gavage (Fig. 1A). It has been previously shown that obese db/db mice failed to develop fibrosis unless they were fed a diet deficient in methionine and choline^15^. The db/db mice, in fact, did not show liver fibrosis as assessed by trichrome staining (Supplementary Fig. 2). Nonetheless, using immunohistochemical (IHC) staining, the expression of α-smooth muscle actin (SMA), the profibrotic protein and an indicator of activated HSCs, was decreased in the liver section of db/db mice after BBR treatment (Fig. 1B). The analysis of histological scoring system for NAFLD activity revealed that the liver histology in the BBR-administered group was significantly improved (Fig. 1C). Consistent with the HE results, BBR treatment was shown to lower the hepatic TG level by 48% (Fig. 1D). Hydroxyproline content, a measure for total collagen, was reduced in the BBR group (Fig. 1E). The serum ALT and AST levels, two markers of hepatic injury, as well as serum TG, TC, LDL, BUN and creatine in db/db mice (Fig. 1F and supplementary Table 1), were reduced by BBR. BBR also showed a significant inhibition in the accumulation of hepatic lipoperoxides (Fig. 1G). We further revealed that BBR reduced cellular TG in oleate acid/palmitate acid (OA/PA)-induced excessive lipid accumulation in cultured HepG2, FAO and primary hepatocytes (Supplementary Fig. 3A–C).

### Effect of BBR on lipogenesis, inflammation, fibrosis and ER stress in db/db mice

**Figure 2:**
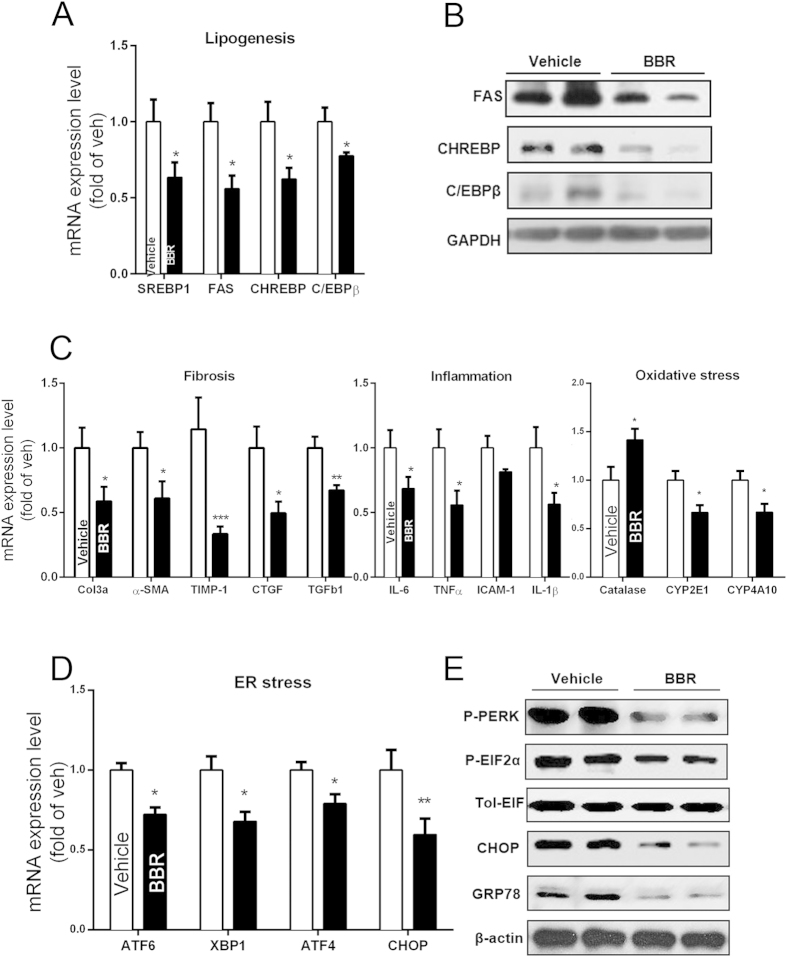
Molecular changes in liver tissue of db/db mice treated with or without BBR. (**A,B**) QPCR and Western blot analysis of liver tissue samples on mRNA (**A**) and protein (**B**) expression of lipogenesis-related genes. (**C**) Analysis of mRNA on hepatic fibrosis, inflammation and oxidative stress-related genes; β-actin was used as an internal control. (**D,E**) Liver mRNA (**D**) and protein (**E**) expression of ER stress-related genes. The data represent the mean ± SEM values. *P < 0.05, **P < 0.01, ***P < 0.001, n = 7–8.

Steatosis is thought to be a prerequisite for NASH and has also been identified as an independent risk factor for liver fibrosis. Our results showed that BBR significantly down-regulated the mRNA levels of SREBP-1c, CHREBP, FAS, and C/EBPβ in db/db mice, all of which are important lipogenesis regulators (Fig. 2A). Consistently, the expression levels of SREBP-1c downstream proteins-CHREBP and FAS, along with the other important transcription factor-C/EBPβ, were significantly down-regulated by BBR (Fig. 2B).

Inflammation has been implicated in the pathogenesis of NASH. Macrophages and monocyte populations are an important source of cytokines in the liver and are key players in NASH progression and resolution^16^. BBR treatment significantly decreased the expression of TNF-α and IL-6 in the liver (Fig. 2C). The IHC results showed that the number of CD68-positive Kupffer cells was significantly decreased after BBR treatment in db/db mice (Supplementary Fig. 4).

It was conceivable that the BBR-mediated reduction in hepatic triglyceride content and inflammation might have protected the mice from liver fibrosis. To address this issue, the mRNA levels of various fibrosis markers were compared. Treating db/db mice with BBR significantly reduced the hepatic expression of transforming growth factor (TGF) β and α-SMA (Fig. 2C). These results showed that BBR also had the potential to prevent the development of fibrosis, although db/db mice cannot develop fibrosis for the genetic background^15^.

The mRNA expression of CYP2E1 and CYP4A10 were downregulated using the BBR treatment (Fig. 2C). CYP2E1 is recognized as a major source of reactive oxygen species and a major mediator of lipid peroxidation^17^,^18^. CYP4A10 assumes the same role as an alternative microsomal lipid oxidase^19^. The mRNA expression of catalase gene, which is an endogenous antioxidant enzyme involved in ROS neutralizing pathways^20^, was upregulated by BBR (Fig. 2C).

ER stress plays an important role in the development of fatty liver disease^21^. To examine whether ER stress signalling is involved in the BBR-ameliorating mechanism on NASH, we investigated the expression levels of critical ER stress markers. We found that the BBR treatment decreased the mRNA expression of ATF6, XBP1, ATF4, and CHOP in the liver of db/db mice (Fig. 2D). Western blot analysis further showed that the protein levels of P-PERK, P-EIF2α, and CHOP were also decreased using the BBR treatment (Fig. 2E).

### BBR-treated MCD diet-fed mice are resistant to liver steatohepatitis and fibrosis

**Figure 3:**
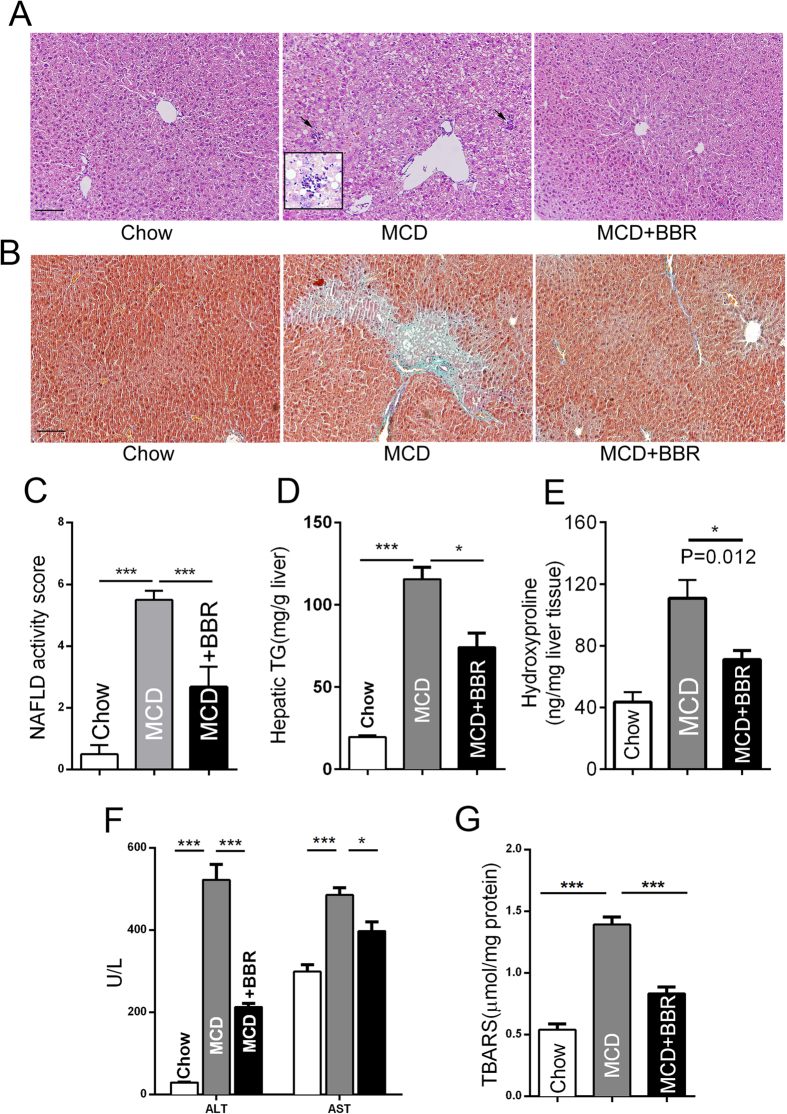
BBR ameliorates MCD diet-induced liver steatosis and fibrosis. Mice were fed a normal chow diet or MCD diet with or without BBR treatment. (**A,B**) Histological analysis of haematoxylin-eosin-stained liver sections (**A**) and collagen deposition evaluated by Masson’s trichrome staining (**B**), magnification: 200×, scale bar, 100 μm. The enclosed box in A shows the enlarged images (400×), and the black arrows indicate inflammatory foci. (**C**) Histopathology analysis was quantified by the NAFLD activity score. The values are mean ± SEM, n = 4 or 3. (**D**) Quantification of hepatic TG contents. (**E**) The hydroxyproline content was measured in the liver of mice. (**F**) Serum levels of ALT and AST. (**G**) Lipoperoxides were measured as described in the Materials and Methods section. The data represent the mean ± SEM values. *P < 0.05, **P < 0.01, ***P < 0.001, n = 7–8.

The MCD diet has been extensively used to produce a diet-induced model of NASH in animals^22^,^23^. Histological analysis using HE staining of the liver sections showed that administering the MCD diet for 8 weeks led to steatosis, inflammation and ballooning degeneration of hepatocytes (Fig. 3A). The NAFLD activity scores were significantly higher in the MCD group than in the control-fed group, whereas BBR markedly reduced the NAS (Fig. 3C). BBR could significantly alleviate liver steatosis, which was confirmed by biochemical analysis of the hepatic triglyceride content (Fig. 3D). BBR had a strong anti-fibrotic effect, which was displayed by Masson’s trichrome staining and hydroxyproline measurement (Fig. 3B, E).

Intake of the MCD diet resulted in prominent increases in ALT, AST (Fig. 3G), and TBARS (Fig. 3G) compared with the chow diet group; However, the accumulation of ALT, AST and lipoperoxides in the MCD group were significantly inhibited after BBR treatment. These results clearly indicate a therapeutic role of BBR on diet-induced NASH by improving the pathological injury in the liver.

Furthermore, BBR decreased cellular TG in MCD medium-induced lipogenesis in AML12 hepatocytes (Supplementary Fig. 3D), which is consistent with our finding in MCD-fed mice.

### Reduction of ER stress, lipogenesis, inflammation, and fibrosis by BBR in MCD diet-fed mice

**Figure 4:**
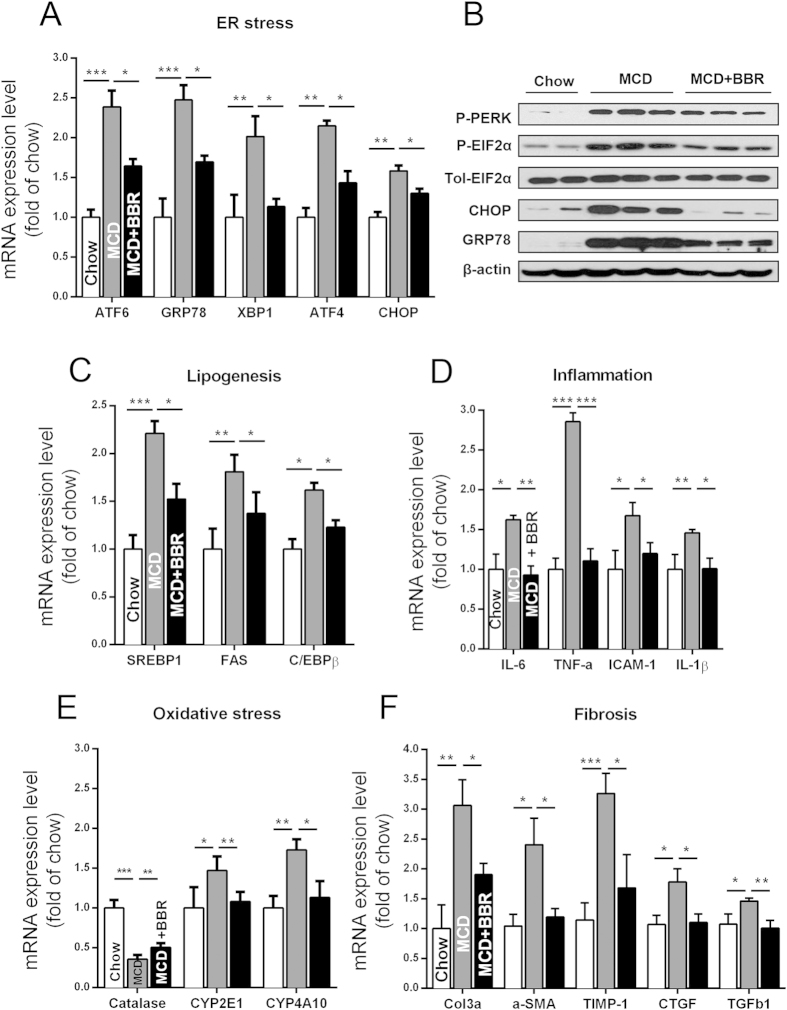
Molecular changes in liver tissue of MCD diet-fed mice treated with or without BBR. (**A,B**) Liver mRNA (**A**) and protein (**B**) expression of and ER stress-related genes. (**C**) QPCR analysis of the liver tissue samples on lipogenesis related genes. (**D–F**) Analysis of mRNA on hepatic inflammation (**D**), oxidative stress (**E**), and fibrosis- (**F**) related genes; β-actin was used as internal control. The data represent the mean ± SEM values. *P < 0.05, **P < 0.01, ***P < 0.001, n = 6–8.

We detected the expression levels of critical ER stress markers. BBR treatment lowered the mRNA expression of ATF6, XBP1, ATF4, and CHOP (Fig. 4A) and decreased the protein levels of P-PERK, P-EIF2α, and CHOP (Fig. 4B). The MCD diet increased the mRNA expression of lipogenesis genes-SREBP-1c, FAS, and C/EBPβ, whereas the BBR treatment could, in part, normalize their expression (Fig. 4C).

BBR markedly repressed the expression of proinflammatory genes. Compared with mice treated with the vehicle, the BBR group showed decreased expression of inflammatory genes in the liver, including TNF-α and IL-6 (Fig. 4D). BBR treatment likely ameliorated hepatic steatosis and fibrosis by protecting the liver from injury caused by lipoperoxide, because the mRNA expression of CYP2E1 and CYP4A10 was downregulated, and the mRNA expression of catalase was upregulated by BBR (Fig. 4E). Consistent with the fibrotic histopathology, hepatic α-SMA, TIMP-1 and TGF-β1 gene expression was decreased after the BBR treatment (Fig. 4F).

### BBR could function as a chemical chaperone and block TM challenge-induced ER stress and NAFLD

**Figure 5:**
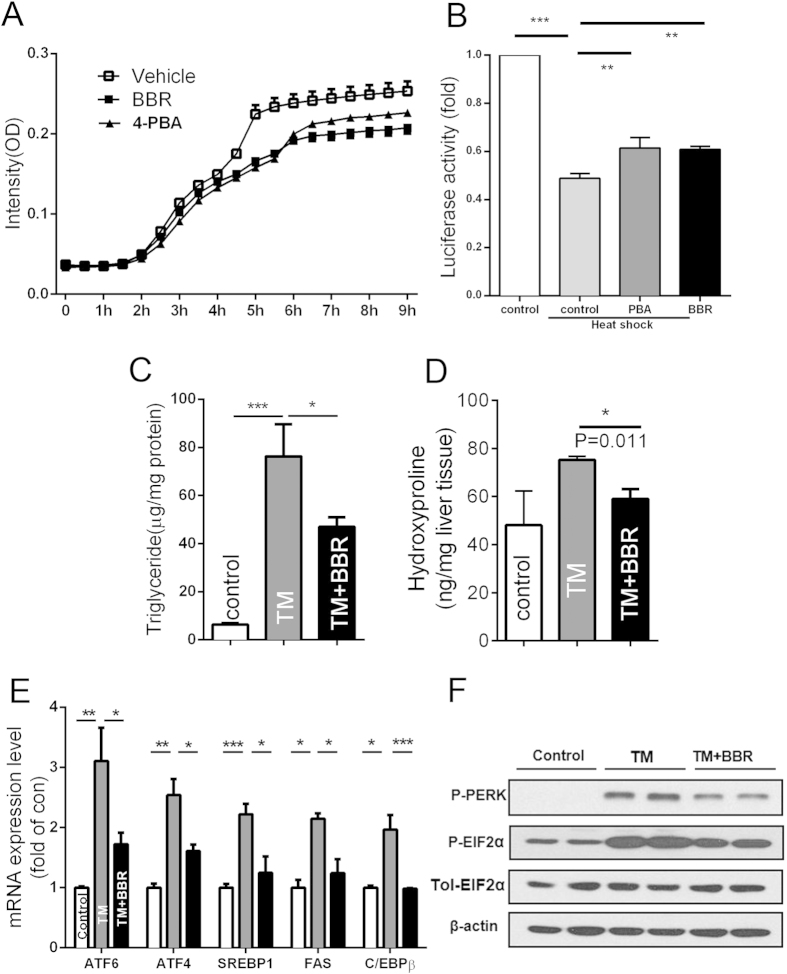
BBR could function as a chemical chaperone and block TM challenge-induced ER stress and NAFLD. (**A**) Chaperone activity of BBR. The rate of aggregation of reduced-α-lactalbumin (r-LA) was measured in the presence or absence of drugs. n = 6. (**B**) *in vivo* refolding assay was performed as described in the Methods section. (**C–F**) C57BL/6J mice were treated with vehicle and BBR for 3 days and then were administered IP TM. After 24 hours, the liver tissues were analysed. (**C**) TG levels were examined in liver tissues. (**D**) The hydroxyproline content was measured. (**E,F**) Liver mRNA (**E**) and protein (**F**) expression of ER stress and lipogenesis-related genes. β-actin was used as internal control. The data are presented as the mean ± SEM values, which represent three independent experiments in triplicate. *P < 0.05, **P < 0.01, ***P < 0.001, n = 7–9.

BBR strongly attenuated ER stress in the two NAFLD/NASH animal models; therefore, we speculated whether BBR may be able to function as a chemical chaperone to reduce ER stress by alleviating protein aggregation. We measured the aggregation of denatured lactalbumin in an *in vitro* system. We observed that BBR markedly attenuated protein aggregation. This effect was stronger than that of 4-phenylbutyrate (4-PBA), which was used as a positive control (Fig. 5A). During another refolding assay, as shown in Fig. 5B, with the addition of 4-PBA and BBR, both compounds could enhance the resolubilization and refolding of the heat-denatured luciferase protein.

To further study the role of ER stress in the BBR-mediated amelioration of NAFLD, a tunicamycin (TM) challenge-induced NAFLD model was used. TM has proven to be an efficient pharmacological tool that induces acute ER stress and fatty liver disease *in vivo.* To study the effect of BBR on ER stress-induced liver pathophysiology, the mice were treated with BBR for 3 days and then challenged with TM through IP injection for another 24 hours. We found excessive hepatic TG and collagen accumulation in the liver of the mice challenged with TM, and we noted pre-treatment with BBR, in part, reversed this effect (Fig. 5C,D). The mRNA expression of SREBP-1c, FAS, C/EBPβ, ATF6 and XBP1 were reduced by BBR (Fig. 5E). A Western blot analysis showed that the protein levels of P-PERK and P-EIF2α were also decreased by BBR (Fig. 5F). The genes related to inflammation and oxidative stress were also dysregulated by TM challenging, and BBR treatment significantly reversed the disturbance (Supplementary Fig. 5A,B). These findings demonstrated that pretreatment with BBR could block the ER stress-induced subsequent reactions, which might be a mechanism mediating BBR’s action in alleviating NAFLD/NASH.

### BBR reverses ER stress-activated lipogenesis through ATF6/SREBP-1c pathway

Our findings showed that BBR treatment improved NASH and attenuated the unfolded protein response (UPR) in animal models. We studied whether the observed amelioration of UPR *in vivo* is due to the direct cell-autonomous effect of BBR on ER stress or an indirect result of a complex *in vivo* interaction among the diverse pathways. When ER stress was induced in HepG2 cells by TM, as expected, the expression of phospho-PERK and phospho-eIF2α were increased; however, subsequent treatment with BBR markedly blocked the expression of the two activated molecules in HepG2 cells (Supplementary Fig. 6). The excessive supply of fatty acids to the liver contributes to hepatic steatosis and ER stress. After treating primary hepatocytes with OA/PA mixture, the mRNA expression levels of ATF6 and SREBP-1c were increased, and BBR treatment could, in part, normalize the increment (Supplementary Fig. 7). These findings suggested that BBR directly ameliorates ER stress.

**Figure 6:**
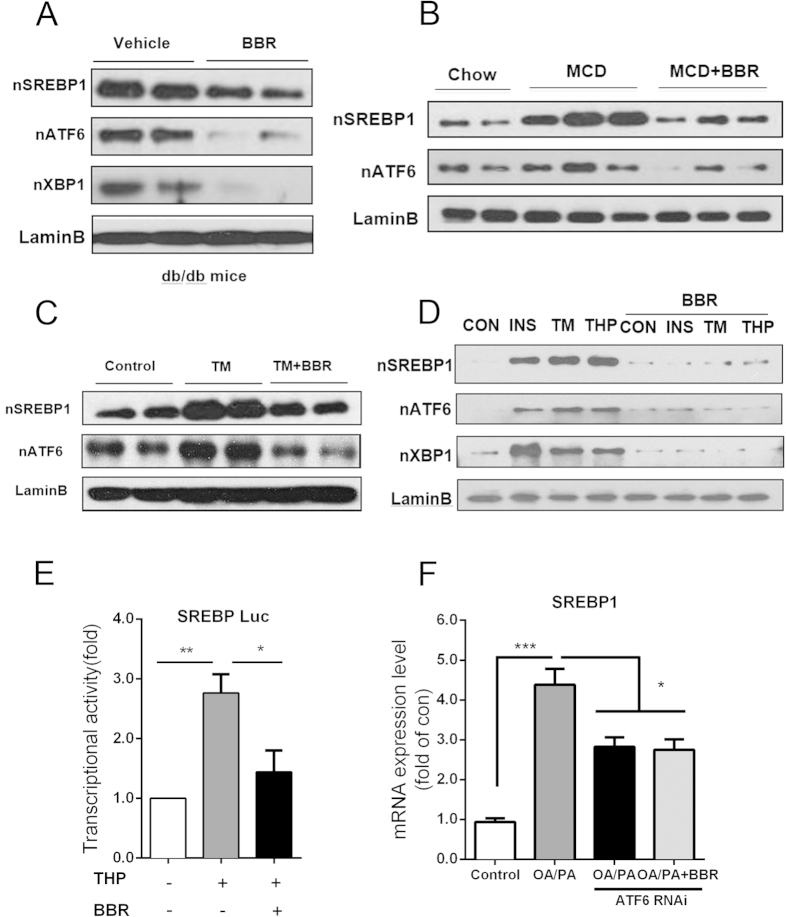
BBR inhibits ER stress activated lipogenesis through ATF6/SREBP-1c pathway *in vitro*. (**A–C**) Western blot analysis of the nuclear accumulation of ATF6, XBP1 and SREBP-1c in the liver tissue samples of db/db (**A**), MCD diet-fed (**B**) and TM-challenged (**C**) mice. Lamin B was used as an internal control. (**D**) Western blot of nuclear SREBP-1c, ATF6, and XBP1 in primary hepatocyte with insulin-, TM-, or THP-induced ER stress, Lamin B as internal control. (**E**) Induction of SREBP-1c promoter activity by THP stimulation was assessed using luciferase reporter assays. (**F**) Regulation of SREBP-1c gene expression by ATF6 knockdown. HepG2 cells were transfected with ATF6 or control RNAi, and were stimulated by OA/PA with or without BBR treatment. The data represent the mean ± SEM values and three independent experiments in triplicate. *P < 0.05, **P < 0.01, ***P < 0.001.

In the liver of db/db, MCD diet-fed and TM-challenged mice, BBR treatment reduced the nuclear accumulation of ATF6 and SREBP-1c (Fig. 6A–C). Additionally, the induction of acute ER stress in primary hepatocytes by insulin, TM, and THA elevated the nuclear accumulation of SREBP-1c and ATF6, whereas BBR abolished such elevation significantly (Fig. 6D). ATF6 is a key transcriptional factor upon the emergence of ER stress, and SREBP-1c is one of the most important nuclear transcription factors that regulates the expression of enzymes for lipogenesis. Therefore, we tested whether ATF6/SREBP-1c was involved in the molecular pathway by which BBR treatment releases ER stress and subsequent lipid accumulation.

ER stress is a major component of the hepatic steatosis^24^. The SREBP-1c promoter luciferase reporter assay showed that THP stimulation-induced ER stress resulted in a more than 2.5-fold induction of SREBP-1c promoter activity and such induction was in part reversed by BBR (Fig. 6E). Both mRNA and western blot assays showed SREBP-1c RNAi silencing, in part, abrogated the fatty acid-mediated induction of SREBP-1c, and BBR could not further decrease this effect. Consistent results were also found in cellular TG content determination experiments (Supplementary Fig. 8A–D). These results clearly showed that SREBP-1c is an important BBR-targeted protein and that BBR could block ER stress-induced upregulation of SREBP-1c and lipid accumulation.

It has been previously shown that a UPR induced by homocysteine is able to activate SREBP-1c and induce lipogenic gene expression^25^. We, therefore, attempted to reason whether ATF6 was involved in BBR’s role in inhibiting the expression of SREBP-1c. As shown in Fig. 6F, RNAi-induced ATF6 depletion (efficiency determined in supplementary Fig. 9), in part, abrogated the fatty acid-mediated induction of SREBP-1c, and BBR could not further decrease the SREBP-1c level. These findings demonstrated that BBR could regulate SREBP-1c through ATF6 at least in the transcriptional level.

## Discussion

Although rodent models of hepatic steatosis and insulin resistance are not always perfectly reflected in the pathology of NAFLD in humans, decreasing TG deposition in the liver is still a potential target for the treatment of NAFLD^26^. Our findings provided conclusive *in vivo* and *in vitro* evidence of BBR’s effect on the reduction of hepatic TG content and support of BBR as a potential agent for NAFLD/NASH patients. Obesity and insulin resistance have been recognized as risk factors for NAFLD and NASH progression^27^,^28^. Insulin resistance was significantly reduced in BBR-treated db/db mice, which may likely exert a certain protective effect against liver steatosis. While hepatic steatosis is commonly considered to be benign, NASH can have serious consequences, such as fibrosis, which is likely to progress to cirrhosis and liver failure, or even to hepatocellular carcinoma. In the present study, BBR halted the progression from steatosis to steatohepatitis and fibrosis in the liver of db/db and MCD diet-fed mice.

Increasing evidence has indicated that the disruption of ER homeostasis, which is often referred to as ER stress or UPR, was found in the livers of patients with NAFLD and obesity^29^,^30^,^31^,^32^. ER stress is a phenotypic hallmark in many liver diseases, including NAFLD, alcohol-induced liver injury, hyperhomocysteinaemia, ischaemia-reperfusion hepatic injury, and chronic hepatitis C as well as hepatitis B and HBV-related hepatocarcinogenesis^33^,^34^,^35^. Our work showed that ER stress in the livers of db/db and MCD diet-fed mice as well as in fatty acid-treated hepatocytes were significantly inhibited by BBR. BBR pretreatment significantly blocked the TM-induced ER stress in the liver of c57 mice and the subsequent increased intrahepatic lipid, hydroxyproline. Our findings suggested that BBR has a strong inhibitory effect on UPR.

SREBP-1c is the master transcription factor for regulating triglyceride synthesis in hepatocytes, which contributes to the pathogenesis of hepatic steatosis^36^. ER stress signalling could increase the transcription and mature nuclear form of SREBP-1c^31^,^24^,^37^, suggesting a critical clue that ER stress leads to the activation of the liver lipogenesis pathway. Although ATF6 and SREBP-1c could be activated by similar mechanisms involving site 1 and 2 proteases^38^, their exact interaction remains unclear. In the present study, we showed that RNAi-mediated knockdown of ATF6 in hepatocytes prevented the inhibitory effect of BBR on fatty acid-induced SREBP-1c expression, which also indicates an upstream role of ATF6 in regulating SREBP-1c. Therefore, our results indicate that BBR could play a critical role in preventing the development of fatty liver by modulating ER stress and dysregulated lipogenesis.

Simple steatosis may progress into steatohepatitis and fibrosis; however, the progression details remain unclear. In recent years, ER stress has been suggested to be one of the most important factors in NAFLD progression, including hepatocyte apoptosis and the activation of non-parenchymal cells, including Kupffer cell and hepatic stellate cells^39^,^40^. Kupffer cells could contribute to the development of liver fibrosis by inducing chronic inflammation^41^, and the activation of HSC may also play a key role during the process in which they could produce and secrete excessive amounts of collagen and extracellular matrix that challenge the cell’s ER folding capacity^39^,^40^. In the present study, BBR shows a robust inhibitory effect on inflammation and fibrosis. However, whether the anti-inflammatory and anti-fibrotic effects of BBR are likely to result from cross-talk between parenchymal and nonparenchymal cells need to be explored in the future.

What is the underlying molecular mechanism of BBR? Chronic metabolic diseases disrupt the folding capacity of proteins in the endoplasmic reticulum. Chemical chaperones consist of a group of low molecular weight compounds that can correct unfolded/aggregated proteins^42^. Our results showed for the first time that BBR exhibited strong chaperone activity by assisting protein folding. Therefore, the therapeutic properties of BBR may, in part, be achieved through a protein-stabilizing action, which subsequently attenuates ER stress.

The accumulation of fatty acids and the generation of an excessive amount of lipid peroxidation products can lead to oxidative stress, which can, in turn, affect fatty acid β-oxidation, cause hepatic steatosis, and produce necroinflammation^18^. Oxidative stress frequently accompanies liver fibrosis and is a well-described determinant of collagen deposition and inflammation^43^,^19^. The marked effects of BBR on reducing intrahepatic lipid peroxidation and the expression of oxidative stress-related genes appear to play another important role in the attenuation of hepatic inflammation and fibrosis.

BBR is a safe therapeutic agent because it has been used for decades in China to treat diarrhoea. Our previous basic research and clinical trial also showed that BBR is safe and effective in treating obese rodents and patients with T2D and dyslipidaemia^9^,^11^,^44^. After oral intake, BBR typically becomes concentrated in the liver rather than in the plasma; therefore, it is suitable for treating NAFLD^45^,^46^. It is indicated that there was a 70-fold increase in the ratio of the area under the concentration-time curve value for BBR (liver versus plasma)^46^.

In summary, the herbal medicine-derived compound, BBR, clearly attenuated liver steatosis and steatohepatitis in experimental mice *in vivo* and in cultured hepatocytes *in vitro*. By functioning as a chemical chaperone targeting ER stress, BBR acts on key pathways associated with NASH pathological progression, including TG accumulation, inflammation, and fibrosis. Our findings supported a beneficial role of BBR and indicated that BBR might be an effective therapeutic strategy against hepatic steatosis and NASH.

## Materials and Methods

### Mouse experiments

Db/db male mice were raised as previously described^9^. From 9 weeks of age, BBR (Sigma-Aldrich, MO, USA) at a dose of 200 mg · kg^−1^ · day^−1^ (BBR group) or an equal volume of vehicle (0.5% methylcellulose, vehicle group) was administered by gavage for 5 weeks (n = 7 per group). After a 12-h fast, all of the mice were euthanised, and their livers and total blood samples were collected for analysis.

For the MCD diet-fed mice, 8 week-old C57BL/6J male mice (Slaccas, Shanghai, China) were used. After an acclimatization period of 1 week, the mice were divided into 3 groups (n = 8 per group). The control group received regular chow, whereas the other MCD and MCD + BBR groups received the MCD diet (Diet Research, NJ, USA). Two weeks after feeding, the mice were treated with BBR or vehicle for another 4 weeks. Thereafter, the mice were euthanised after a 12-h fast, and the liver tissues and blood samples were collected for analysis.

In TM (Merck, NJ, USA) experiments, the C57BL/6J mice at 8 weeks were acclimated for 1 week and divided into 3 groups (n = 10 per group) and then treated using BBR or vehicle for 3 days and subsequently were injected IP with TM (1 mg/kg body weight) or vehicle control (150 μM dextrose). After 24 h post TM injection, the liver tissues and blood samples were collected for evaluation. All of the experimental procedures involving the use of animals were conducted in conformity with PHS policy and approved by the Animal Use and Care Committee of Shanghai Jiao Tong University.

### Histopathology analysis

The formalin-fixed liver tissue was processed, and 5-μm-thick paraffin sections were stained with haematoxylin and eosin (H&E) and Masson’s trichrome for histological analysis. The histological examination was performed using the histological scoring system for NAFLD by an experienced pathologist without prior knowledge of the treatments. The NAFLD activity score (NAS) was quantified by summing the scores of steatosis (0–3), lobular inflammation (0–2), and hepatocellular ballooning (0–2). NASH was defined in the cases of NAS of ≥5^47^,^48^,^49^. Immunohistochemistry approaches were performed as previously described^50^, using a rabbit anti-CD68 (Abcam) and rabbit anti-α-SMA (Abcam).

### TG measurement

For the determination of lipids, the homogenates from the cells or liver tissues were extracted using a methanol-chloroform mixture according to the Folch method^51^. The triglyceride content of each sample was measured after evaporation of the organic solvent using the triglyceride measurement reagent (Sigma-Aldrich) adhering to the manufacturer’s instructions.

### Assays for hepatic lipoperoxides

The lipid peroxidation was determined by quantifying the amount of thiobarbituric acid reactive substances (TBARS) according to a previous description with several modification^23^. The liver tissues were sonicated in 200 μl RIPA buffer, after centrifugation (3000 rpm, 10 min); 100 μL of the supernatant was obtained for the reaction with 200 μl 10% trichloroacetic acid (TCA); TBARS (Sigma-Aldrich) were subsequently measured with 1,1,3,3-tetramethoxypropane as a standard (Sigma-Aldrich).

### Hydroxyproline measurement

The hydroxyproline content in the liver tissue was measured as previously described with several modifications^52^. The sample absorbance was measured at 560 nm in duplicate. Purified hydroxyproline (Sigma-Aldrich) was used to set a standard. The hydroxyproline content was expressed in nanograms of hydroxyproline per milligram liver.

### Cell culture

Isolation and culture of C57BL/6J mice primary hepatocytes were previously described^53^. HepG2 and FAO cell lines were grown in DMEM supplemented with 10% foetal bovine serum, 1% L-glutamine and 1% P/S (Invitrogen, NY, USA). The cells were sub-cultured in DMEM containing 0.2% bovine serum albumin (BSA) (Invitrogen, NY, USA) for 24 h until the cultures reached 70% to 80% confluence. The medium was replaced with DMEM containing BSA-conjugated OA/PA at concentrations of 0.5 mM and 1 mM, respectively, with or without BBR (5 μM), or 5% BSA as control for an additional 24 hours. Immortalised mouse normal hepatocytes (AML12) were cultured as previously described^23^. PA, OA, TM, and thapsigargin (THP) were obtained from Sigma-Aldrich; insulin was provided by Novartis (NJ, USA).

### Chaperone activity

Chaperone activity using α-lactalbumin aggregates was measured as previously described with several modifications^54^. Aggregation was monitored in the presence or absence of reagents, such as BBR, sodium 4-phenylbutyrate (4-PBA, positive control), by measuring turbidity at 488 nm using a microplate reader (BioTek, USA). The aggregation of r-LA was induced by BSA aggregates. Aggregation gradually increased in a time-dependent manner after the addition of denatured BSA (0–9 h at 37 °C).

*In vivo* refolding assay was performed as previously described with minor modifications^55^,^56^. HepG2 hepatocytes in triplicate were incubated in DMEM containing 0.02% BSA for 12 hours, and co-transfected with pCytLuc (B2.28: luciferase expression construct) and pCMV-Hsp90 plasmid. After 24 hours, BBR (5 μM) and PBA (20 mM) were added to the medium for a 24-hour treatment. Before and during the heat shock experiments, the medium was removed, and 20 μg/ml cycloheximide and 20 mM 4-morpholinepropanesulfonic acid (MOPS) (pH 7.0) were added to the medium. For luciferase activity measurements, triplicate samples were obtained, and the cells were lysed and assayed using the Promega luminometer system.

### SREBP-1c promoter construct, transfection, and luciferase assays

SREBP-1c promoter luciferase plasmid was constructed according to a previous publication^57^. Transfections were performed using Lipofectamine 2000 reagent (Invitrogen, NY, USA). HepG2 hepatocytes in triplicate were incubated in DMEM containing 0.02% BSA for 12 hours and co-transfected with 2 μg of SREBP luciferase reporter plasmid and 0.005 μg of a control plasmid phRL-TK encoding the synthetic *Renilla* luciferase gene (Promega) for each 24-well plate well. After 24 hours, BBR (5 μM) and THP (5 μg/ml) were co-added to the medium and treated for 24 hours. Luciferase activity was measured and normalized following the manufacturer’s instructions.

### Preparation for nuclear and cytoplasmic protein extracts

The nuclear and cytoplasm proteins were extracted from the liver tissues using NE-PER nuclear and cytoplasmic extraction reagents (Thermo Scientific, NH, USA). The protein concentration was determined using the BCA protein assay kit (Thermo Scientific, NH, USA).

### Quantitative PCR

The total RNA and protein extracts were isolated from the hepatocyte lysates or mouse livers for gene expression analysis. A quantitative PCR was performed using cDNA transcribed from total RNA according to the manufacturer’s instructions.

### Western blot analysis

Western blotting was performed using primary antibodies against phosphorylated PERK, phosphorylated EIF2α, EIF2α, CHOP, C/EBPβ, GAPDH, β-actin (Cell Signaling, MA, USA), ATF6 (Abcam, Maharashtra, India), FAS (BD Bioscience, NJ, USA), SREBP-1c (Thermo Scientific, NH, USA), carbohydrate-responsive element-binding protein (CHREBP) (Novus Biologicals, NJ, USA), and LaminB (Santa Cruz Biotechnology, CA, USA). Western blots were developed using ECL Western blotting substrate and quantified using ImageJ software (for results, see Supplementary Fig. 10).

### SREBP-1c and ATF6 silencing

HepG2 cells were transfected with RNAi duplexes via RNAi MAX Lipofectamine reagent (Invitrogen, NY, USA). SREBP-1c was silenced in cells with ON-TARGET plus siRNAs SMARTpool (L-006891-00-0005) and parallel cells with ON-TARGET plus Non-targeting Pool (D-001810-10-05) at a concentration of 10 nM (Thermo Scientific). ATF6α was silenced in cells with duplex siRNAs (HSS177036) at a concentration of 10 nM (Invitrogen). Parallel cell cultures were transfected with equal concentrations (10 nM) of Stealth negative universal control (Invitrogen). At 24 hours after transfection, the medium was replaced with complete growth medium containing OA/PA (1 mM) and BBR (5 μM).

### Statistical analysis

All of the data are presented as the mean ± SEM values. Between-group comparisons were performed using Student’s *t*-test. The comparisons among multiple groups were performed using one-way ANOVA with post hoc test (Tukey’s Multiple Comparison Test). P < 0.05 was considered to be statistically significant.

## Additional Information

**How to cite this article**: Zhang, Z. *et al.* Berberine prevents progression from hepatic steatosis to steatohepatitis and fibrosis by reducing endoplasmic reticulum stress. *Sci. Rep.* **6**, 20848; doi: 10.1038/srep20848 (2016).

## Supplementary Material

Supplementary Information
