# Supplementary material for: Berberine prevents progression from hepatic steatosis to steatohepatitis and fibrosis by reducing endoplasmic reticulum stress
Source: Sci Rep. 2016 Feb 9;6:20848. doi: 10.1038/srep20848 (PMC4746620; doi:10.1038/srep20848)
Supplement: Supplementary Information [file srep20848-s1.pdf]

TITLE PAGE

Title: Berberine prevents progression from hepatic steatosis to steatohepatitis and fibrosis by reducing endoplasmic reticulum stress

Short title: BBR treats NAFLD/NASH by suppressing ER stress

Zhiguo Zhang[1,*], Bo Li[1, 2, 4*], Xiangjian Meng[1, 3*], Shuangshuang Yao[1*], Lina Jin[1], Jian Yang[1], Jiqiu Wang[1], Huizhi Zhang[1], Zhijian Zhang[1], Dongsheng Cai[2], Yifei Zhang[1], Guang Ning[1]

[1]Shanghai Institute of Endocrine and Metabolic Diseases, Shanghai Clinical Center for Endocrine and Metabolic Diseases, Department of Endocrinology and Metabolism, China National Research Center for Metabolic Diseases, Ruijin Hospital, Shanghai Jiao Tong University School of Medicine, Shanghai 200025, China

[2]Department of Molecular Pharmacology, Albert Einstein College of Medicine, Bronx, New York 10461, USA

[3] Department of Endocrinology, Yijishan Hospital of Wannan Medical College, Wuhu, Anhui Province 241000, China.

4 Department of Endocrinology, Xinhua Hospital, Shanghai 200092, China

[*]These authors contributed equally as first authors.

Correspondence and requests for materials should be addressed to Y.-F.Z. (Email: feifei-a@163.com)

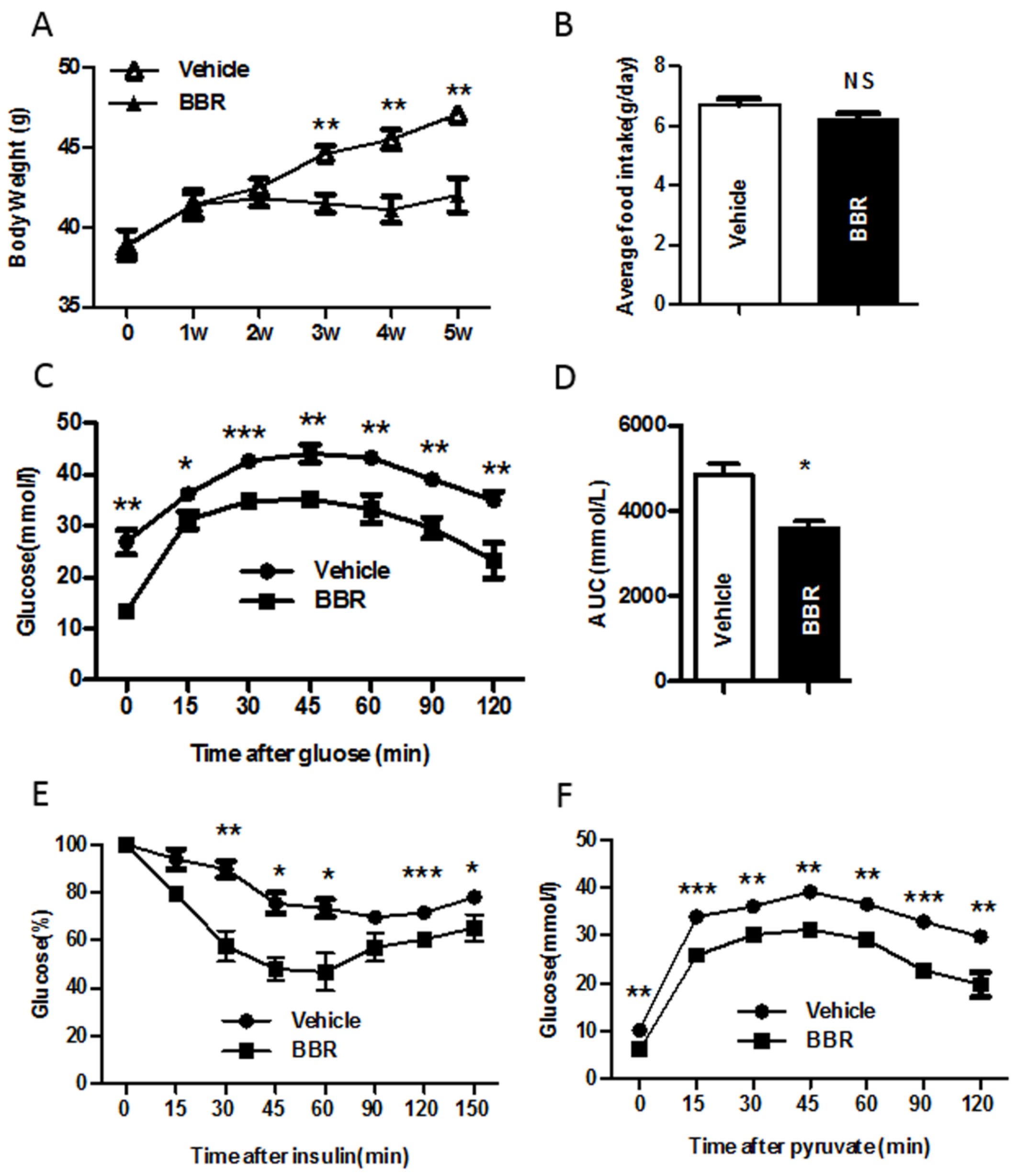

Supplementary figure legends:

Supplementary Figure 1. BBR reduces obesity and improves insulin sensitivity.

A: Body weight evaluation of db/db mice treated with vehicle or BBR every 3 day (g) (n = 8). B: average food intake (n = 8). C-D: Intraperitoneal glucose tolerance test on db/db mice treated as in (A) and injected with 0.75 g glucose/kg after overnight fasting (n = 6-8) (C). The adjacent bar graph (D) represents the average area under the curve (n =7- 8). E: Insulin tolerance test was evaluated through and injected with 1 U

insulin/kg after 6 hr of fasting (n = 7-8). F: Pyruvate tolerance test was evaluated on db/db mice treated as in (A) and injected with 1 g pyruvate/kg after overnight fasting (n = 7-8). Values represent means ± SEM. Error bars represent SEM, and significant differences compared to vehicle controls are indicated by *P<0.05, **P<0.01, ***P < 0.001

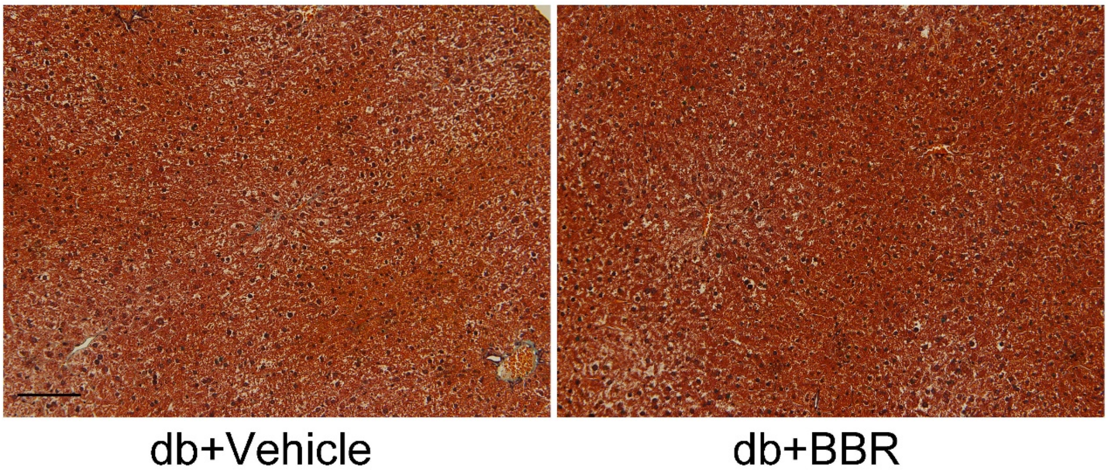

Supplementary Figure 2. collagen deposition evaluated by Masson's trichrome staining (X200), scale bar, 100 μ m.

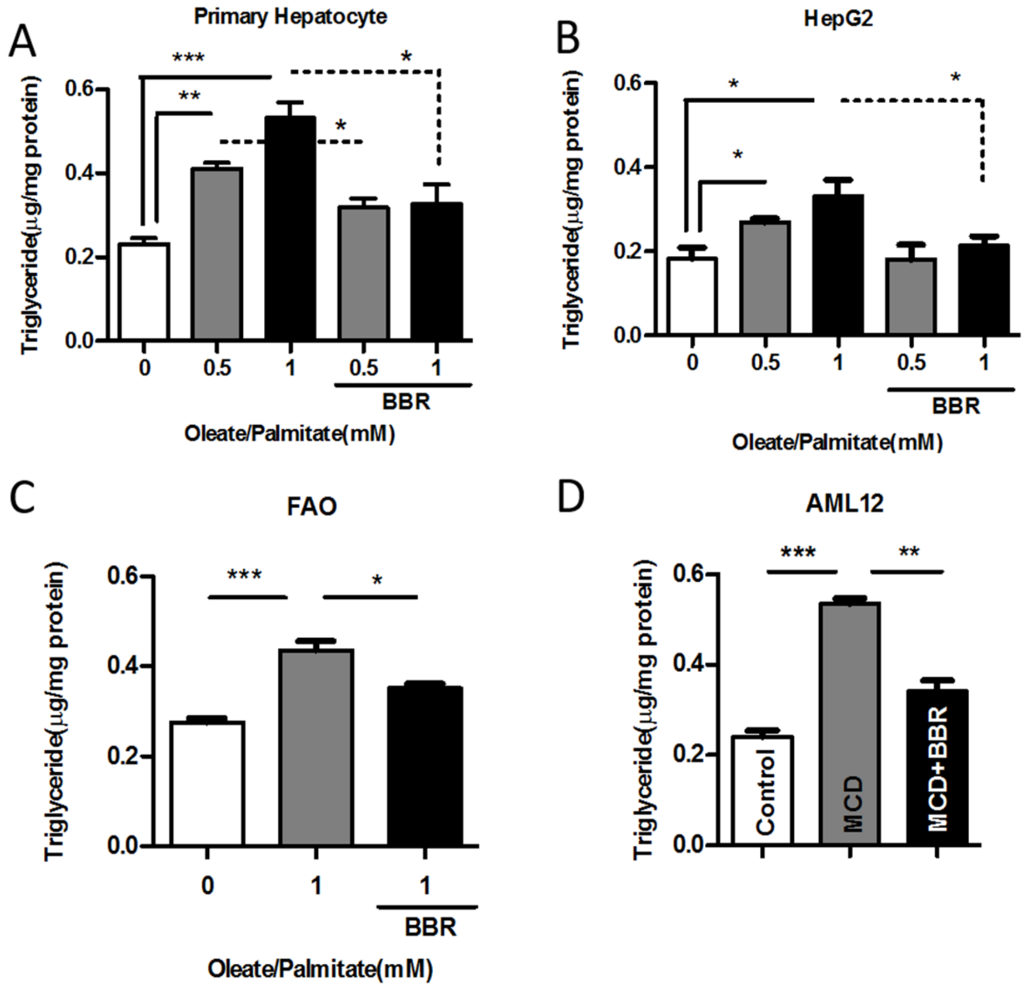

Supplementary Figure 3. BBR decreased cellular TG accumulation in different hepatocytes. A-C: Lipid accumulation levels in OA/PA induced steatosis in primary hepatocytes, HepG2, and FAO cell lines. D: Lipid accumulation level in MCD medium incubation-induced steatosis in AML12 cells. Data are presented in mean ± SEM that represent three independent experiments in triplicate. *P < 0.05, **P < 0.01, ***P<0.001.

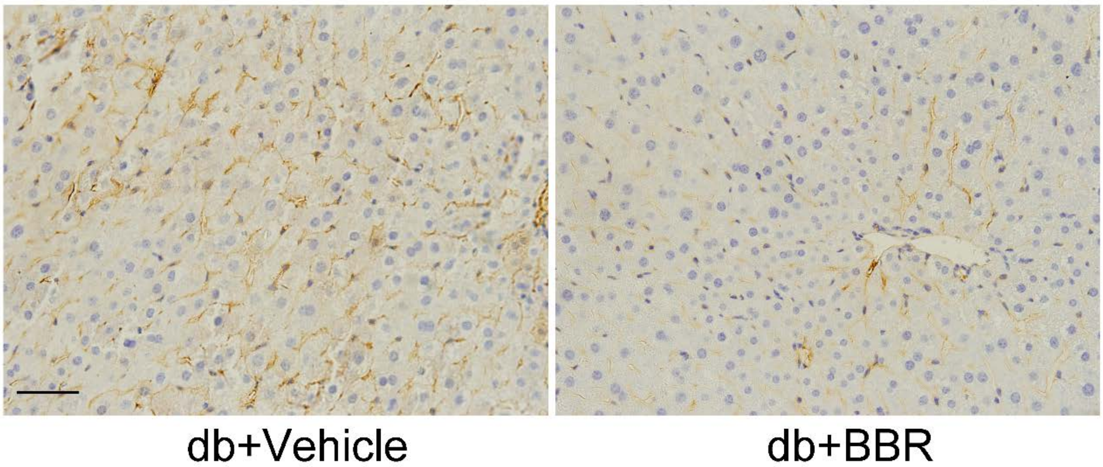

Supplementary Figure 4. Immunohistochemistry for CD68 (brown stain) in liver sections of db/db mice (X400), scale bar, 50 μ m.

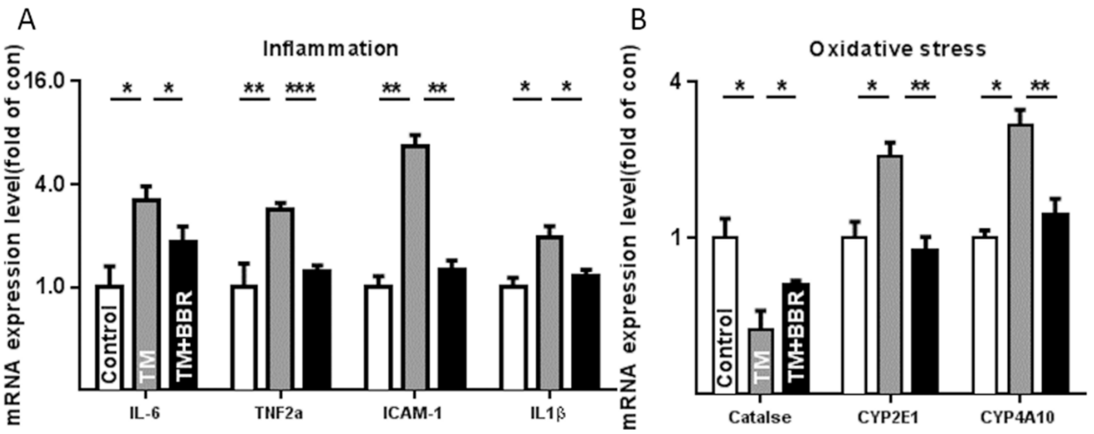

Supplementary Figure 5. BBR could function as a chemical chaperone and block TM challenge-induced ER stress and NAFLD. C57BL/6J mice were treated with vehicle and BBR for 3 days and then gave I.P. TM. After 24 hours, the liver tissues were analyzed. A-B: Analysis of mRNA on hepatic inflammation (A) and oxidative stress (B) related genes, β-actin was used as internal control. Data are presented in mean ± SEM that represent three independent experiments in triplicate.*P < 0.05, **P < 0.01, ***P<0.001, n=6-8.

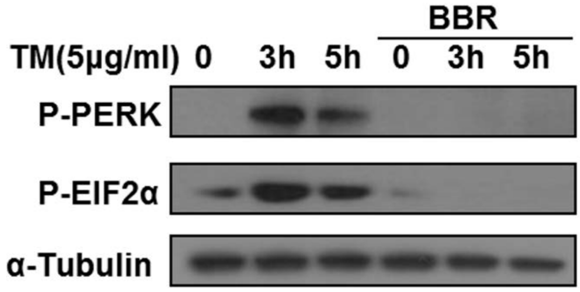

Supplementary Figure 6. Western blot of P-PERK, P-EIF2α in HepG2 cell line stimulated with TM for a different time in the presence or absence of BBR, α-Tubulin as internal control.

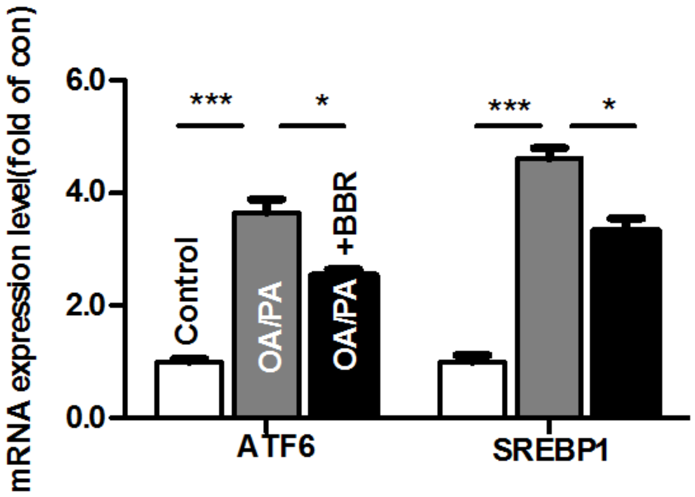

Supplementary Figure 7. ATF6 and SREBP1 mRNA expression level by incubation of 1mM OA/PA in primary hepatocytes with or without BBR treatment. Data are presented in mean ± SEM that represent three independent experiments in triplicate.*P <0.05, **P < 0.01, ***P<0.001.

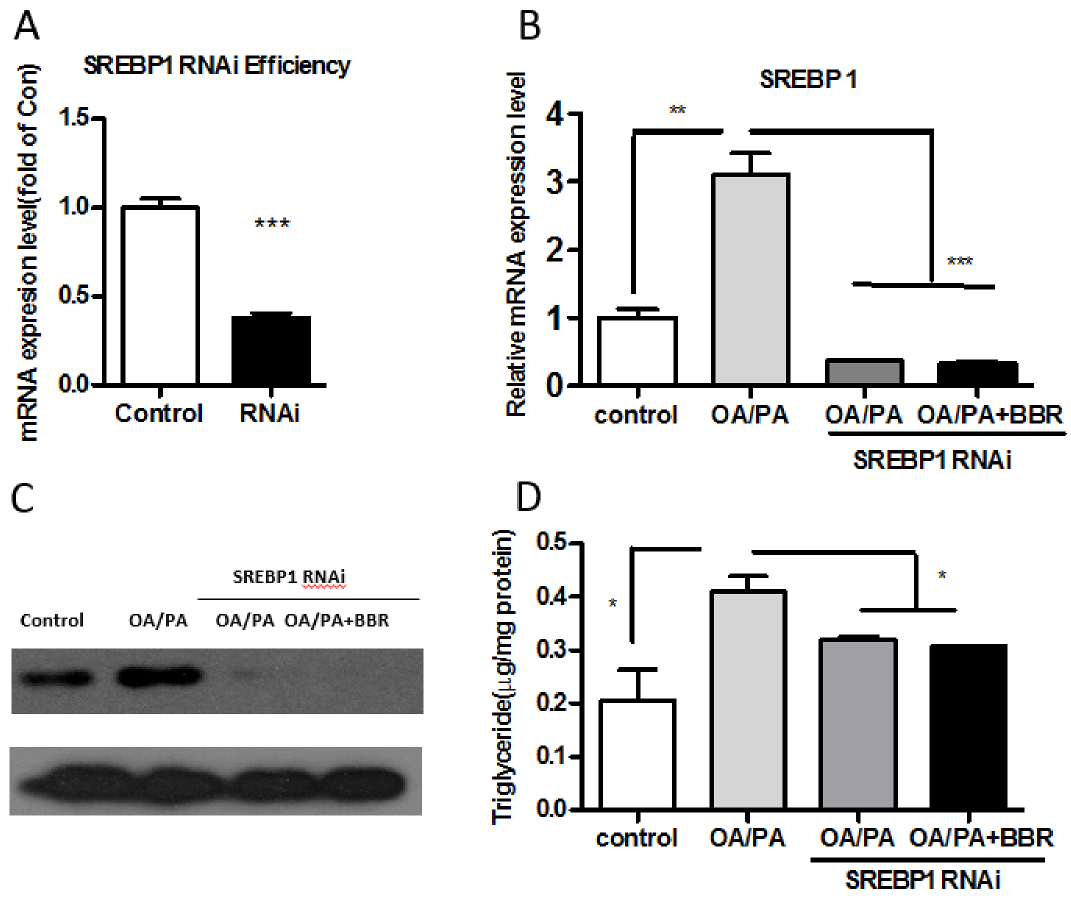

Supplementary Figure 8. SREBP-1c knockdown blocked the TG reduction by BBR treatment in HepG2 cells A-B: HepG2 cells were transfected with SREBP1 or control RNAi, and were stimulated by OA/PA with or without BBR treatment. Efficiency of SREBP-1c RNAi-mediated knockdown in HepG2 cells was verified by qPCR (A), the collected samples were analyzed for SREBP-1c mRNA (B) and protein levels (C), as well as cellular TG content (D) as previously described. Data are presented in mean ± SEM that represent three independent experiments in triplicate.*P <0.05, **P < 0.01, ***P<0.001.

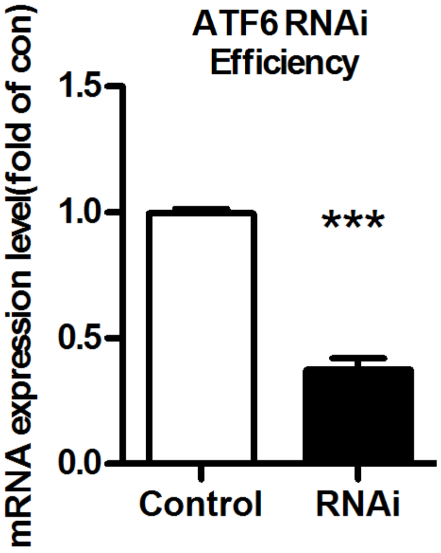

Supplementary Figure 9. Efficiency of ATF6 RNAi-mediated ATF6 knockdown in HepG2 cells. Data are presented in mean ± SEM that represent three independent experiments in triplicate.*P <0.05, **P < 0.01, ***P<0.001.

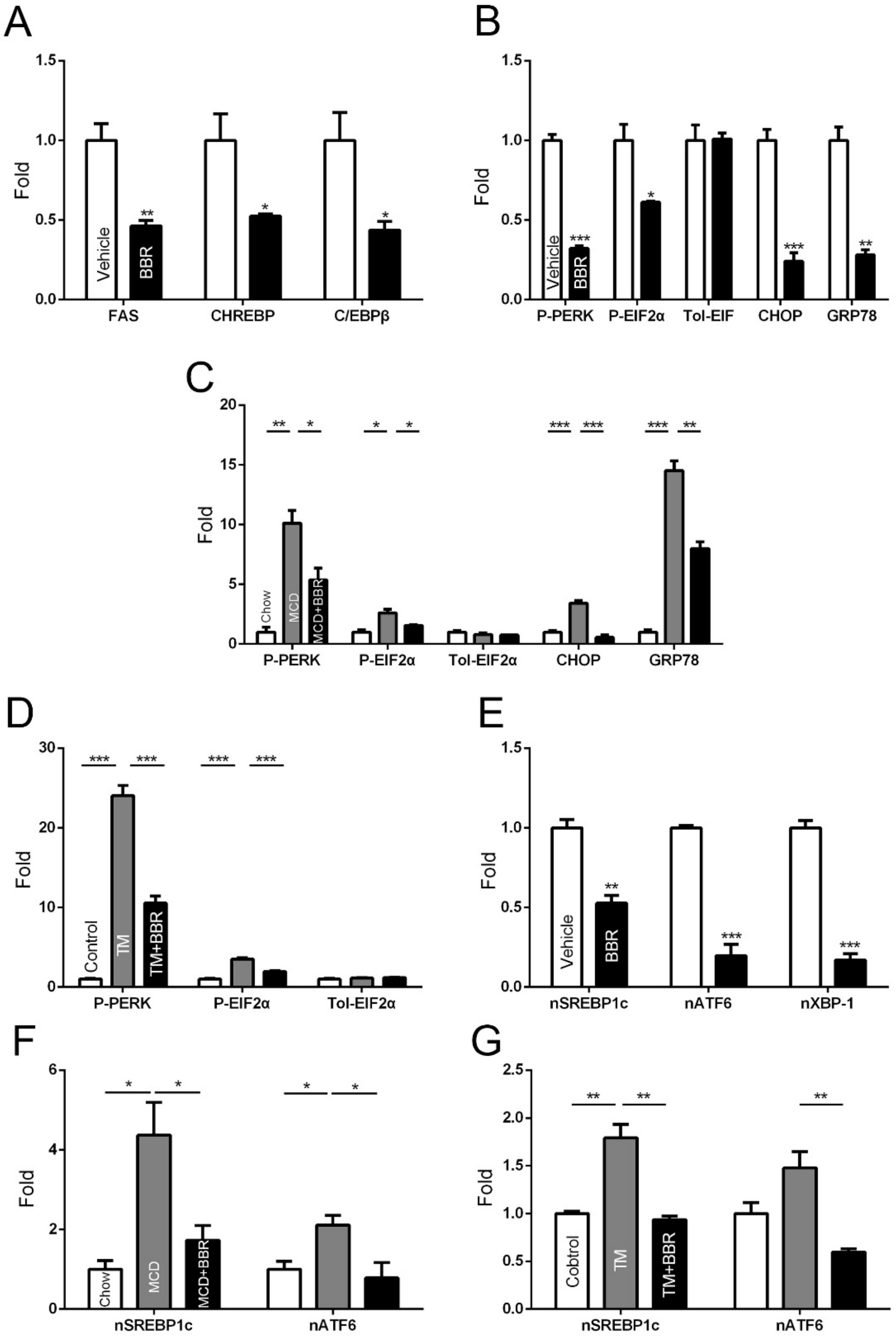

Supplementary Figure 10. Results of Western blots were quantified by Image J software. A. quantified result of Fig. 2.B; B. quantified result of Fig. 2E; C. quantified result of Fig. 4B; D. quantified result of Fig. 5B; E. quantified result of Fig. 6A; F. quantified result of Fig. 6B; G. quantified result of Fig. 6C.

Supplementary Table1

General parameters of BBR treatment for 5 weeks in db/db mice

Parameters	Vehicle	BBR
Serum TG(mmol/L)	4.82 ± 0.18	3.55 ± 0.15 a
Serum TC(mmol/L)	4.36 ± 0.21	3.78 ± 0.14 a
Serum HDL(mmol/L)	2.69 ± 0.12	3.08 ± 0.09 a
Serum LDL(mmol/L)	0.75 ± 0.045	0.56 ± 0.059 a
Serum BUN(mmol/L)	9.463 ± 0.42	8.10 ± 0.45
Serum Cr(μmol/L)	19.92 ± 0.37	17.16 ± 0.62 a
Serum FFA(mEq/L)	1.691 ± 0.063	1.435 ± 0.091 a

Data are presented as mean ± SEM (n = 8).

TG, total triglyceride; TC, total cholesterol; HDL, high-density lipoprotein;

LDL, low-density lipoprotein; BUN, blood urea nitrogen;

Cr, cretinine; FFA, free fatty acid.
